# Comparison of functional outcome and patient satisfaction between patients with socket prosthesis and patients treated with transcutaneous osseointegrated prosthetic systems (TOPS) after transfemoral amputation

**DOI:** 10.1007/s00068-022-02018-6

**Published:** 2022-06-18

**Authors:** Marcus Örgel, Frederik Schwarze, Tilman Graulich, Christian Krettek, Friederike Weidemann, Horst-Heinrich Aschoff, Marcel Winkelmann, Alexander Ranker

**Affiliations:** 1 Trauma Department, Hannover Medical School (MHH), Carl-Neuberg-Straße 1, 30625 Hannover, Germany; 2 Department of Physical Medicine and Rehabilitation, Hannover Medical School (MHH), Carl-Neuberg-Str. 1, 30625 Hannover, Germany; 3 Orthopedic, Trauma and Sportsmedicine Department, KRH Klinikum Agnes Karll Laatzen, Hildesheimer Straße 158, 30880 Laatzen, Germany

**Keywords:** Rehabilitation, Transfemoral amputation, Transcutaneous osseointegrated prosthetic systems, Socket prosthesis, Osseointegration

## Abstract

**Purpose:**

The aim of this retrospective analysis was to investigate and evaluate differences in functional outcome and satisfaction of patients treated with a TOPS and patients using socket prosthesis after transfemoral amputation.

**Methods:**

This retrospective comprehensive analysis included patients from a single hospital, and was conducted between February 2017 and December 2018. Overall *n* = 139 patients with prosthesis were included and divided into two comparable groups (socket- and TOPS group). Incomplete data sets were excluded. This led to *n* = 36 participants for the socket- and *n* = 33 for the TOPS group. Functional outcome and satisfaction were evaluated by Patient Reported Outcome Measures (PROMs). The used PROMs were: Questionnaire for Persons with a Transfemoral Amputation (Q-TFA), EQ5D-5L, Satisfaction with Prosthesis Questionnaire (SAT-PRO), Prosthesis Mobility Questionnaire (PMQ 2.0) and Functional Independence Measure (FIM).

**Results:**

Significant results in favor of TOPS patients were identified for the EQ-5D 5L (*p* = 0.004), Q-TFA (*p* = 0.000), SAT-PRO (*p* = 0.000) and PMQ 2.0 (*p* = 0.000). For FIM, no statistical significance was found (*p* = 0.318).

**Conclusion:**

In this study, transfemoral amputees treated with an osseointegrated prosthetic attachment (TOPS) showed significantly higher scores for mobility and satisfaction. This demonstrates the high potential of TOPS in the prosthetic treatment of patients with transfemoral amputation with regard to their functional abilities in daily life.

**Supplementary Information:**

The online version contains supplementary material available at 10.1007/s00068-022-02018-6.

## Introduction.

### Background

Vascular diseases such as arteriosclerosis and diabetes mellitus, as well as tumors, accidents and war injuries are reasons for limb amputations [1–4]. Lower limb amputations (LLA) account for approximately 75% of all amputations (41.8% transtibial and 34.5% transfemoral) [5]. Early mobilization after LLA reduces complications such as pain or phantom sensation, edema, muscle atrophy or contracture. It improves maintenance of postural reflexes, and has also significant functional and psychological benefits leading to improved acceptance of the prosthetic fitting [6, 7]. Transcutaneous osseointegrated prosthetic systems (TOPS), of which endo–exo prosthetics (EEP) are a part, are a suitable alternative to conventional socket prostheses [8–10]. Usually, TOPS are used in such cases, where conventional socket-suspension-systems are not possible. This happens if the stump is very short, or show high-volume changes or skin irritations due to sweating and pressure and also if the shape of the stump is morphologically not suitable for the socket. Nevertheless, TOPS offer good functional outcomes and high levels of satisfaction [11–13]. These studies predominantly compare the same cohort of patients before and after using TOPS [12, 14–20]. Thus, TOPS are still seen as an alternative option if socket-suspension-systems fail. This view is also often supported the supposed high infection risk, which frequently results in soft-tissue infections and must be addressed via oral antibiotics or minor surgery. In addition, the studies have shown that the infections do not affect the implant lifetime [13, 21–24]. Therefore, it is meaningful to investigate whether TOPS-user and socket-suspension-system-user differ in functional outcome and satisfaction with their device. We hypothesize that TOPS patients are significantly more satisfied than patients with socket prostheses regarding their rehabilitation outcome.

This could put the limited value of TOPS as an optional application after transfemoral amputation in a different light.

## Methods

### Study design and size, setting and participants

An observational study was performed with PROMs in an outpatient clinic of a university hospital in northern Germany.

From February 2017 to December 2018, we retrospectively assessed the rehabilitation results of two groups—TOPS and socket user—after transfemoral amputation, using a structured interview. The surgery for TOPS patients was performed by two surgeons (senior and junior surgeon) at the same hospital where the data were collected. Inclusion criteria were the completely data sets and transfemoral amputation. Exclusion criteria were the incompletely answered questionnaires and amputations other than transfemoral.

For this purpose, five questionnaires were utilized: Questionnaire for Persons with a Transfemoral Amputation (Q-TFA), EQ5D-5L, Satisfaction with Prosthesis Questionnaire (SAT-Pro), Prosthesis Mobility Questionnaire (PMQ) and Functional independence measure (FIM). We used a separate questionnaire for assessing the socio-cultural prerequisites for rehabilitation and the rehabilitation results of the two cohorts.

**Fig. 1:**
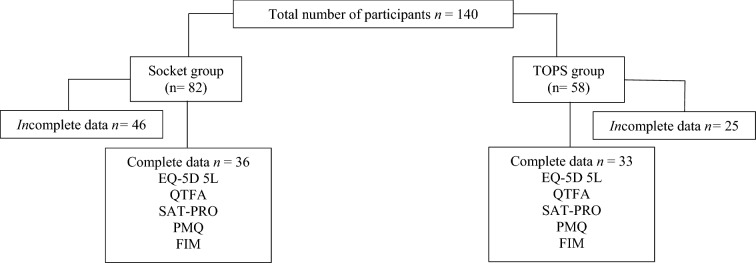
Cohort distribution. *n* = number of participants

This study followed the “Strengthening the Reporting of Observational Studies in Epidemiology (STROBE)” reporting guideline. Data collection was performed in an outpatient clinic of a university hospital. All data were collected by the same person. If any problems occurred, an independent expert study nurse assisted participants in filling out the questionnaires. There was no time limit for completing the questionnaires. Between February 2017 and December 2018, 140 patients could be included in this study. All participants with incomplete data sets were excluded. Detailed information is shown in Fig. 1.

### Questionnaires

The descriptive data contain demographic data such as age, sex and BMI [kg/m^2^], as well as side of amputation, the reason for amputation as well as information about the socio-cultural circumstances (Table 1). Five types of PROMS were handed out, which are briefly explained in the following lines.

**Table 1:** Demographic data of the whole cohort and socio-cultural prerequisites for rehabilitation

	Socket group (*n* = 36)	TOPS group (*n* = 33)	Total (*n* = 69)	*p* Value
Sex *n (%)*^*##*^				
Male	18 (50)	17 (51.5)	35 (50.7)	0.9^c^
Female	18 (50)	16 (48.5)	34 (49.3)	
Side *n (%)*^*##*^				
Left	18 (50)	20 (60.6)	37 (53.6)	0.32^c^
Right	18 (50)	13 (39.4)	32 (44.9)	
Reason for amputation *n (%)*^*##*^				
Trauma	23 (63.9)	21 (63.6)	44 (63.8)	0.84^d^
Tumor	3 (8.3)	3 (9.1)	6 (8.7)	
Vascular disease	4 (11.1)	4 (12.1)	8 (11.6)	
Sepsis	2 (5.6)	1 (3.0)	3 (4.3)	
Iatrogenic	3 (8.3)	4 (12.1)	7 (10.1)	
Missing data	1 (2.8)		1 (1.4)	
Age [years] mean ± SD *(95%-CI)*^*##*^	48.6 ± 13.0	52.1 ± 9.7	50.3 ± 11.7	0.44^b^
	(43.9–53.2)	(44.5–53.1)	(47.7–52.9)	
BMI [kg/m^2^] mean ± SD *(95%-CI)*^*##*^	26.9 ± 5.2	29.5 ± 6.5	28.3 ± 5.7	0.15^a^
	(25.1–28.8)	(27.1–31.8)	(27.0–29.7)	
CCI [%] mean ± SD *(95%-CI)*^*##*^	89.6 ± 10.4	93.9 ± 5.3	91.26 ± 12.0	0.22^a^
	(85.8–93.3)	(85.4–93.1)	(88.6–93.9)	
Months since TOPS treatment mean ± SD *(95%-CI)*	–	30.5 ± 41.5		
		(21.7–47.1)		
Reduction in employment *n (%)*^*##*^				
Yes	11 (36.1)	18 (54.5)	29 (42.0)	0.5^a^
No	13 (30.6)	15 (45.5)	28 (40.6)	
No data	12 (33.3)	–	12 (33.3)	
Get back to work *n (%)*^*##*^				
Yes	19 (52.8)	21 (63.6)	40 (58.0)	0.9^a^
No	3 (8.3)	11 (33.3)	14 (20.3)	
No data	14 (38.9)	1 (3.0)	15 (21.7)	
Current employment *n (%)*^*##*^				
Employed	13 (36.1)	16 (48.5)	29 (42.0)	0.8^a^
Retired/OAP	11 (30.6)	15 (45.5)	26 (45.6)	
Unemployed	–	2 (6.1)	2 (2.9)	
No data	12 (33.3)	–	12 (17.4)	
Changes of the working situation after the amputation *n (%)*^*##*^				
No	5 (13.9)	15 (45.5)	20 (29.0)	0.2^a^
Yes	18 (50.0)	15 (45.5)	33 (47.8)	
No data	13 (36.1)	3 (9.1)	16 (23.2)	
Hours worked per week before amputation^*##*^ mean ± SD *(95%-CI)*	43.3 ± 6.9	37.0 ± 10.0	39.8 ± 9.5	0.2^a^
	(39.0–47.7)	(31.5–42.5)	(35.9–43.7)	
Hours worked per week after amputation^*##*^ mean ± SD *(95%-CI)*	35.8 ± 12.4	29.0 ± 10.9	31.4 ± 12.1	0.1^a^
	(28.0–43.7)	(23.0–35.0)	(26.4–36.4)	
Nursing care *n (%)*^*##*^				
Yes	7 (19.4)	12 (36.4)	19 (27.5)	0.6^a^
No	17 (47.2)	21 (63.6)	38 (55.1)	
No data	12 (33.3)	-	12 (17.4)	
Degree of nursing care *n (%)*^*##*^				
1°	1 (2.8)	2 (6.1)	3 (4.3)	0.8^c^
2°	4 (11.1)	7 (21.2)	11 (15.9)	
3°	2 (5.6)	3 (9.1)	5 (7.2)	
No data	29 (80.6)	21 (63.6)	50 (72.5)	
Degree of disability (%)^*##*^	82.7 ± 13.9	86.1 ± 12.2	80.0 ± 14.1	0.09^d^
	(76.3–88.9)	(81.7–90.4)	(74.2–85.8)	
Help with basic daily care *n (%)*^*##*^				
Outpatient nursing				0.8^d^
Service	2 (5.6)	1 (3.0)	3 (4.3)	
Family members	5 (13.9)	8 (24.2)	13 (18.8)	
No external help	17 (47.2)	24 (72.7)	41 (71.9)	
No data	12 (33.3)	–	12 (17.4)	
Mental health problems *n (%)*^*##*^				
Yes	11 (30.6)	11 (33.3)	22 (31.9)	0.3 ^c^
No	13 (36.1)	22 (66.7)	35 (50.7)	
No data	12 (33.3)	–	12 (17.4)	
Satisfaction with Prosthesis *n (%)*^*##*^				
Not at all satisfying	5 (13.9)	1 (3.0)	6 (8.7)	0.000^a^*
Rather not satisfying	7 (19.4)	2 (6.1)	9 (13.0)	
Moderately satisfying	4 (11.1)	1 (3.0)	5 (7.2)	
Rather satisfying	4 (11.1)	4 (12.1)	8 (11.6)	
Absolutely satisfying	4 (11.1)	25 (75.8)	29 (42.0)	
No data	12 (33.3)	–	12 (17.4)	

*BMI* Body Mass Index, *CCI* Charlson Comorbidity Index, *SD* STANDARD DEVIATION, *CI* confidence interval

^a^Mann–Whitney-*U*-test

^b^*t*-Test

^c^Pearson’s Chi-squared-test

^d^Fisher’s exact test

^*^p < 0.05

^##^non-normally distributed data

### Questionnaire for persons with a Transfemoral Amputation (Q-TFA)

The Q-TFA is a PROM published in 2004 that was developed for transfemoral amputees using socket or osseointegrated prosthesis [25]. A common problem with many PROMS is ceiling effects, which make it difficult to reliably measure and distinguish between very high mobility and excellent mobility. This score was developed to be able to differentiate between these points. The questionnaire is very comprehensive and tests several areas. These are prosthesis use, mobility, problems in daily life and general health/quality of life. A maximum of 100 points can be obtained as the best rehabilitation result. The Q-TFA was validated in 2004 by Hagberg et al. on 156 transfemoral amputees with a socket prosthesis [25]. A German version of this PROM was used.

### European Quality-of-Life 5 Dimensions 5 Level Version (EQ5D-5L)

The EQ5D-5L is a common quality-of-life questionnaire. It was developed by the EuroQoL group in 1996 [26]. In its original form, it consists of a visual analogue scale and five questions on mobility, self-care, usual activities, pain or discomfort and anxiety or depression, each with three possible answers. There are 3^5^ = 243 combinations of levels, each of which can be described with a five-digit number [26, 27]. The EQ5D is usually used for patient groups. However, there are also studies with samples of the general population [27–32]. There were several ceiling effects with high frequencies of the best response pattern [29, 31], so that a modified questionnaire was developed that retains the five domains but expands the number of possible answers from three to five, the so-called EQ-5D-5L [33, 34]. The EQ5D is frequently utilized in evaluating the health-related quality of life (HRQoL) in patients with LLA and available in 169 languages [35]. The German version was used [36].

### Satisfaction with Prosthesis Score (SAT-PRO)

The SAT-PRO was developed to determine the satisfaction of people with lower limb amputations and their prosthesis. It contains 15 questions about satisfaction with the prosthesis in everyday life, which are answered on a four-point Likert scale (0–3 Points). The maximum score is 45, whereby the result shall be converted into percentages (0–100% satisfaction) [37]. The translated and validated German version was used [38].

### Prosthesis Mobility Questionnaire 2.0

The PMQ is a questionnaire with 12 questions about mobility in everyday life, which are answered using a five-point Likert scale [39, 40]. In a Rasch analysis by Burger et al. it became apparent that it seems to make more sense to include only those questions into the total score which are associated with greater difficulty (e.g., it is difficult for me to go upstairs or to go down stairs) [40]. Thus, the new PMQ 2.0 has the same questions as the PMQ, and differs only in counting and adding to the total score (max. score 40 = best result = very good mobility). It was translated by Ranker et al. in line with the respective guidelines in 2020 and checked for quality criteria [41].

In a further study, it was shown that using the Rasch-Analysis, the PMQ2.0 can distinguish more precisely between people with high mobility abilities than the well-known and often used LCI-5[42].

### Functional independent measure (FIM)

The FIM can be utilized for quality assurance, therapy monitoring, and for classifying patients considering their functional disability [43]. Kidd et al. [44] confirmed the validity of FIM in a comparative validity and reliability study in 1995. In this study, the FIM-Short questionnaire was used for the subgroup’s cognition and motor skills. In each case, the best result was 91 points. This corresponds to complete independence of the patient. The FIM was validated on samples with LLA [45–47] and translated in German followed by the validation [48].

### Self-designed questionnaire

Finally, a self-designed questionnaire was handed out, which was intended to ascertain the socio-cultural conditions for rehabilitation on the one hand and the results of the rehabilitation of the two cohorts with regard to financial status, need for care, psychological comorbidities and quality of life on the other hand. The following areas were analysed: professional status before and after the amputation as well as the satisfaction with the fitted prosthetic system, degree of disability, degree of care, previous mental illnesses and influence of the prosthesis on the quality of life.

### Statistical methods

Statistical analysis was performed using SPSS 26 (IBM, SPSS Inc., Chicago, IL). After checking for normal distribution, Student’s *t*-test was used for normal and Mann–Whitney-*U*-test for non-normal variables. The significance value was set to *p* < 0.05. The baseline data were descriptively analysed and are given in percentage ratios.

## Results

**Fig. 2:**
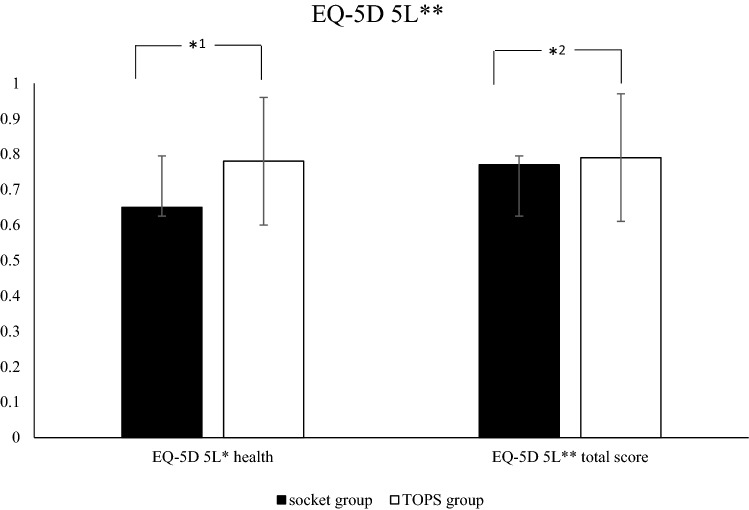
Results of the EQ-5D 5L for the socket and TOPS group. **EQ-5D 5L: European Quality-of-Life 5 Dimensions 5 Level Version, *^1^*p*  <  0.004, *^2^*p*  <  0.035

**Fig. 3:**
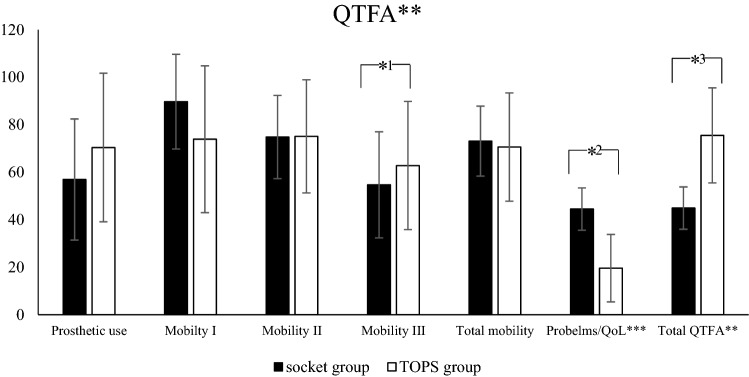
Results of the Q-TFA for the socket and TOPS group. **QFTA: Questionnaire for Persons with a Transfemoral Amputation, ***QoL: Quality of life *^1^*p* < 0.009, *^2^*p* < 0.000, *^3^*p* < 0.000

**Fig. 4:**
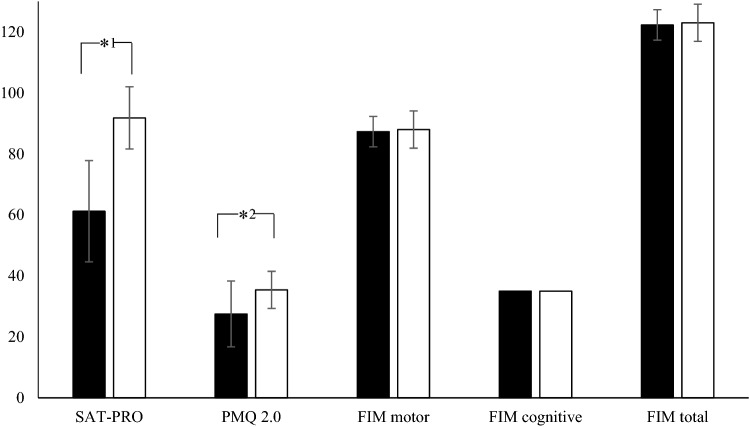
Results of the questionnaire SAT-PRO, PMQ, and FIM for the socket and TOPS group. SAT-PRO: Satisfaction with the prosthesis, PMQ 2.0: Prosthesis mobility questionnaire 2.0, FIM: Functional Independent Measure *^1^*p* < 0.000, *^2^*p* < 0.000

Demographic data of the cohort as well as the results of the questionnaires are shown in Table 1 and Figs. 2, 3 and 4 The demographic data such as age, sex, cause of amputation, BMI, etc. show no statistically significant difference between the groups and prove therefore their comparability. In terms of satisfaction with a single question (“Are you satisfied regarding your prosthesis”), 75.8% of the people with a TOPS answered with the highest possible answer (“absolutely satisfied”).

## Discussion

### Key results

According to our hypothesis, the resulting differences between both groups could be proven for mobility and satisfaction with exoprosthetics with significantly higher satisfaction in TOPS patients. For the questionnaires EQ-5D 5L (*p* = 0.004), Q-TFA (*p* = 0.000), SAT-PRO *p* = 0.000), and PMQ 2.0 (*p* = 0.000), we were able to identify significant results in favor of TOPS patients. FIM (*p* = 0.318) was the only group for which we could not find a significant difference.

### Interpretation

The fact that the PMQ results are better in the TOPS group shows that satisfaction is related to mobility—the more mobile a patient is, the happier s/he is. Wurdemann et al. emphasised this aspect in their work and provided in their study evidence of a strong positive correlation between mobility and both quality of life and general satisfaction [49]. Furthermore, it could be possible that different "locomotive ability" causes a bias. Kark et al. showed that after lower limb amputation, physical, mental, and social functioning is more relevant than mobility [50].

Nevertheless, the FIM has been developed for stroke patients and the tasks are very easy. This could lead to ceiling effects, so that the distinction at the upper “borders” of the results is not possible. This could be the cause why the FIM showed just minimal greater autonomy and independence as well as less use of aids in daily life in the TOPS group without presenting a significant difference. The cognitive part of the FIM showed no difference between the two groups, so that worse results in the motor part cannot be attributed to cognitive deficits such as lack of understanding of questions or instructions for behavior. Despite the lack of significant difference between the two groups, the FIM is a suitable instrument to evaluate rehabilitation progress for comparisons. Leung et al. and Karmaker et al. used the FIM to objectify their examinations of amputee patients [45, 51–54].

The significant difference in the PMQ 2.0 shows that the FIM was probably not the best measurement to distinguish between the mobility-levels of the two groups. Thus, PROM showed lower ceiling effects in validation studies and is useful for people with higher mobility grades [41, 42]. Therefore, the higher values in PMQ2.0 in the TOPS group can be interpreted as an advantage of TOPS. This coincides with other results in the literature, where TOPS users show high mobility grades [13, 19, 55–57]. Furthermore, the higher mobility level could possibly influence the higher level of satisfaction in this group.

Our self-designed questionnaire showed a significant difference between the “impact of the prosthesis on quality of life” and the “degree of disability” between both groups. No significant difference could be found in the pursuit of an occupation after transfemoral amputation for both cohorts. Furthermore, the average working hours reported for both cohorts did not differ from those reported by the Federal Statistical Institute for all employed persons in Germany [58].

Comparing our Q-TFA results with other studies also shows significantly improved outcome in favor of TOPS patients. However, some of these studies present longitudinal instead of cross-sectional results [12–14, 17–20, 25, 57, 59, 60]. Similarly, our results for the EQ-5D 5L show results comparable to other authors. A study by Cutti et al. in 2016, in which 127 transfemoral amputees, who were fitted with two different socket prosthesis systems, were surveyed, shows scores for the EQ5D-5L that are slightly higher than the results for the socket group in our study [61]. Comparisons with other studies are only possible with the EQ5D-3L questionnaire. Therefore, the EQ5D-5L seems to be more sensitive and able to reduce ceiling effects [62]. In addition to higher scores, Pospiech et al. showed no statistically significant difference in EQ-5D-3L scores in cohorts of 17 socket prosthesis patients and 22 TOPS patients, although the number of cohorts must be taken into account in the interpretation of the results [20].

SAT-PRO also shows significant differences between the two groups. In contrast to the SF-36, the SAT-PRO is rarely used in the literature. There is only one study that included SAT-PRO to compare life quality and functionality of patients using socket prothesis with bilateral versus unilateral lower extremity amputations. This study showed no significant difference in rehabilitation outcomes between unilateral and bilateral amputees [63].

Demographic data (see Supplement Table 2) of in- and excluded participants slightly differ in terms of gender distribution. Excluded patients are predominantly male. Additionally, excluded patients have been treated with TOPS longer ago and are slightly older. However, both observations are not significant. There were no differences in terms side of amputation, reason of amputation, body mass index, as well as Charlson comorbidity index. To what extent these differences influenced our results remains unclear. So far, the literature has demonstrated only gender-independent advantages of TOPS in comparison to socket prostheses [11, 13, 17, 19, 55, 57, 64–68]. We are not able to determine whether this is due to high or low satisfaction with TOPS. From our clinical experience, the vast majority of TOPS patients would never switch back to a socket system despite minor difficulties.

### Strengths and limitations

The high percentage of excluded data due to incomplete information should be critically noted. Many patients often did not fill out the entire questionnaires. This circumstance was because some of the study participants only visited the outpatient clinic once. They returned the incomplete questionnaire, so that the missing information only became apparent during the anonymous retrospective analysis. We then no longer had the opportunity to complete the data. Moreover, it is a retrospective study with a small collective, even though it is also a large number of cases for this topic compared to the current literature. Furthermore, the data inconsistency of the descriptive data concerning the socio-cultural sector should also be highlighted. This inconsistency could be explained by the fact that these are discrete and personal topics on which not every participant wanted to give information. A further limitation of the study is the study design itself. It is well known that prospective study designs offer much more value. However, this dataset was created retrospectively. Nevertheless, it contains important results about the satisfaction of patients treated with TOPS. To the best knowledge of the authors, no data on the patient’s perceived satisfaction with a comparable high sample size have been published yet. The importance of the study design is enhanced by the big sample size for such a particular research field as the transcutaneous osseointegrated prosthetic system.

## Conclusion

In this study, transfemoral amputees treated with an osseointegrated prosthetic attachment (TOPS) showed statistically significant higher scores for mobility and satisfaction. This demonstrates the high potential of TOPS in the prosthetic treatment of patients with transfemoral amputation with regard to getting on daily life and its positive impact on their quality of life.

## Supplementary Information

Below is the link to the electronic supplementary material.

Supplementary file1 (DOCX 15 KB)
