# Supplementary material for: Comparison of functional outcome and patient satisfaction between patients with socket prosthesis and patients treated with transcutaneous osseointegrated prosthetic systems (TOPS) after transfemoral amputation
Source: Eur J Trauma Emerg Surg. 2022 Jun 18;48(6):4867–76. doi: 10.1007/s00068-022-02018-6 (PMC9712408; doi:10.1007/s00068-022-02018-6)
Supplement: Supplementary file 1 — Supplementary file1 (DOCX 15 KB) [file 68_2022_2018_MOESM1_ESM.docx]

**Table 2** Supplement demographic data of excluded participants

|  | Included | Excluded | p-Value |
| --- | --- | --- | --- |
| Sex *n (%)^##^*  Male  Female | 34  35 | 23  5 | 0.003 |
| Side *n (%)^##^*  Left  Right | 37  32 | 14  16 | 0.517 |
| Reason for amputation *n (%)^##^*  Trauma  Tumor  Vascular disease  Sepsis  Iatrogenic | 44  6  8  3  7 | 18  4  1  0  7 | 0.207 |
| Age [years] mean ± SD *(95%-CI)^##^* | 50.3 ± 11.7  (47.7 – 52.9) | 55.1 ± 14.0  (49.9 – 60.3) | 0.096 |
| BMI [kg/m^2^] mean ± SD *(95%-CI)^##^* | 28.2 ± 6.0  (26.7 – 29.7) | 28.3 ± 4.3  (26.6 – 30.0) | 0.520 |
| CCI [%] mean ± SD *(95%-CI)^##^* | 91.9 ± 8.3  (89.9 – 93.9) | 84.6 ± 21.3  (76.7 – 92.6) | 0.493 |
| Months since TOPS treatment mean *±* SD *(95%-CI)* | 30.5 ± 41.5  (15.7 – 45.2) | 50.3 ± 49.1  (21.9 – 78.7) | 0.066 |
